# Unilateral Tongue and Lip Edema: A Rare Presentation of Angiotensin-Converting Enzyme (ACE) Inhibitor-Induced Angioedema

**DOI:** 10.7759/cureus.101033

**Published:** 2026-01-07

**Authors:** Gonçalo Carneiro, Iara Ferreira, Catarina Reigota

**Affiliations:** 1 Internal Medicine, Unidade Local de Saúde de Entre Douro e Vouga, Santa Maria da Feira, PRT; 2 Internal Medicine, Centro Hospitalar e Universitário de Coimbra, Coimbra, PRT

**Keywords:** ace inhibitor-induced, bradykinin-mediated angioedema, drug-related side effects, tongue angioedema, unilateral angioedema

## Abstract

Angiotensin-converting enzyme (ACE) inhibitor-induced angioedema is a rare but potentially life-threatening adverse effect that may occur years after treatment initiation. Although it typically presents with bilateral facial or oropharyngeal swelling, unilateral tongue involvement is exceptionally rare and diagnostically challenging. We report the case of a 77-year-old man on long-term ACE inhibitor therapy who presented with sudden unilateral tongue and lip edema. Symptoms improved with supportive treatment and drug discontinuation. This case highlights the importance of recognizing atypical presentations to ensure prompt diagnosis, appropriate management, and improved patient safety.

## Introduction

Angiotensin-converting enzyme (ACE) inhibitor-induced angioedema is considered a rare but potentially life-threatening adverse effect of this class of antihypertensive drugs. The overall incidence of ACE inhibitor-induced angioedema is relatively low (reported between approximately 0.1% and 0.7%) [1-4], but accounts for a significant proportion of drug-induced angioedema in the emergency setting (17-40%) [4,5], reflecting its clinical impact [4,5].

Most cases present with diffuse or bilateral swelling; however, unilateral involvement - especially of the tongue - is exceptionally rare, with only a limited number of case reports in the literature documenting this atypical pattern [2,6,7].

Distinguishing unilateral ACE inhibitor-induced angioedema from other acute tongue pathologies relies heavily on clinical suspicion and a careful review of medication history. Recognizing this atypical presentation is crucial, as delayed diagnosis may lead to unnecessary interventions or missed opportunities for early airway protection. Early discontinuation of the offending agent and supportive management remain the cornerstones of treatment [1]. Reporting this case of unilateral tongue involvement is particularly important given its extreme rarity and the limited number of similar cases in the literature.

## Case presentation

**Figure 1:**
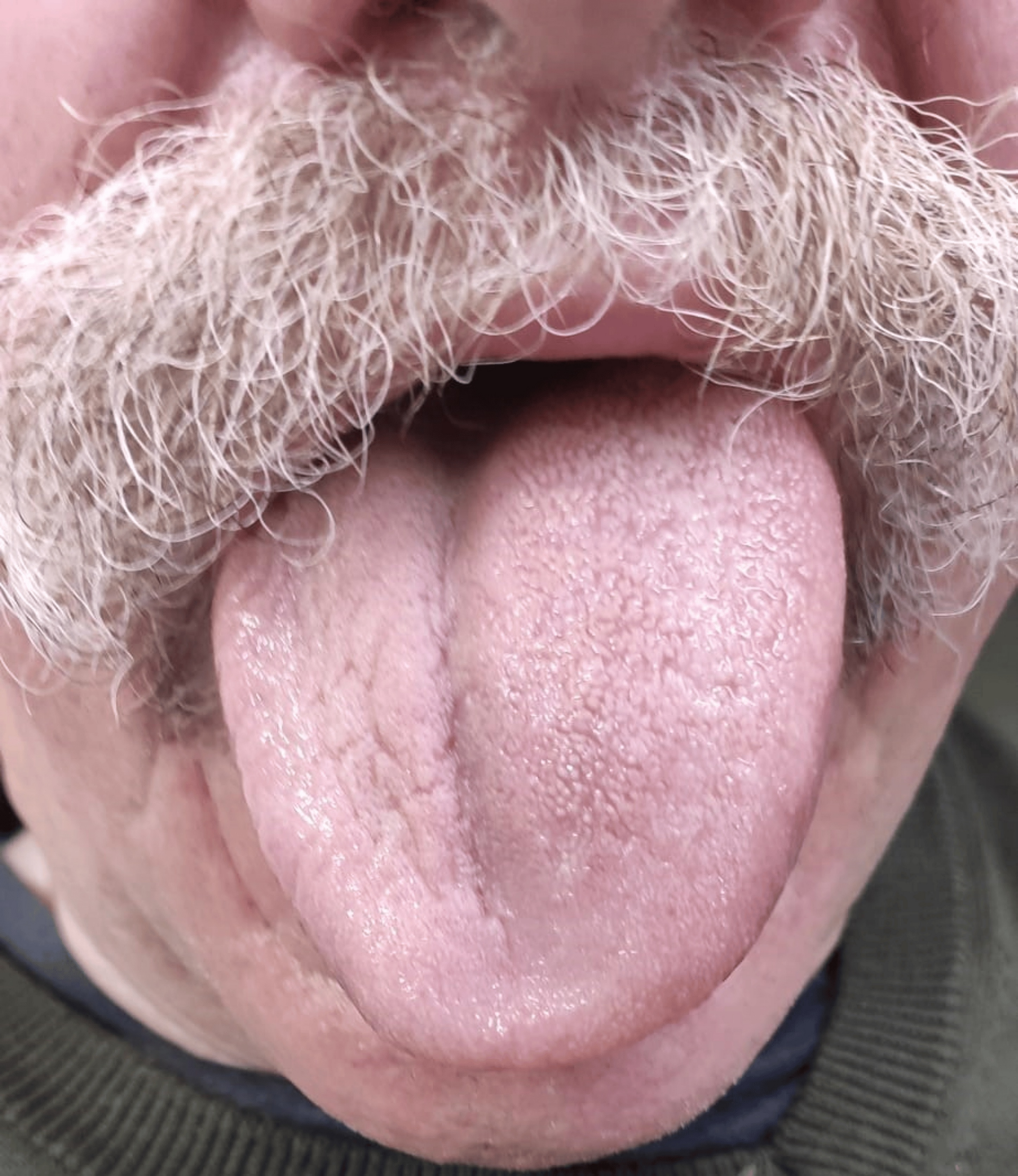
Unilateral tongue edema induced by na angiotensin-converting enzyme inhibitor. Image taken and published with the patient’s informed consent.

A 77-year-old man with a history of hypertension, who had been treated with an ACE inhibitor for more than 20 years, presented to the Emergency Department with sudden-onset unilateral swelling of the left side of the tongue and lower lip (Figure 1), which had begun three hours prior to presentation. The patient denied recent trauma (including insect bites), exposure to new foods or medications, and reported no personal or family history of allergies or urticaria. Vital signs were stable, including hemodynamic status and peripheral oxygen saturation. Physical examination revealed no edema of other anatomical structures, no signs of airway compromise, and no associated cutaneous rash. Laboratory investigations - including complete blood count, electrolytes, renal function, liver enzymes, and C-reactive protein - were within normal limits.

Within a few hours of receiving intravenous fluids, corticosteroids, and antihistamines, the patient showed marked improvement in the edema. As no alternative cause was identified, the ACE inhibitor was discontinued, and therapy was switched to an angiotensin receptor blocker. The patient was subsequently discharged with referral for follow-up with his family physician and an immunoallergy consultation, which occurred six months later. During this interval, the patient reported no recurrence of angioedema, and blood pressure control remained within acceptable limits under the new therapeutic regimen. A subsequent follow-up visit three months later included repeat laboratory testing, including complement levels, all of which were within reference ranges. Based on the clinical presentation, response to treatment, and sustained resolution following drug discontinuation, ACE inhibitor-induced angioedema was considered the most likely diagnosis. The patient continues routine outpatient follow-up without further complications.

## Discussion

Although rare, ACE inhibitor-induced angioedema remains a clinically important adverse effect due to the widespread use of these medications (particularly for hypertension, chronic kidney disease, and heart failure) and the risk of airway obstruction in more severe presentations [1,2,4]. Although the reaction is thought to result from impaired degradation of bradykinin [1-4], leading to increased vascular permeability and localized tissue swelling, definitive evidence for an exclusive bradykinin-mediated mechanism is lacking [4]. Consequently, angioedema may occur both early in the course of treatment and after prolonged exposure, even several years after therapy initiation [1-4].

Unilateral tongue edema represents an exceptionally rare and diagnostically challenging presentation [2,6,7]. Case reports indicate that most documented unilateral tongue angioedema occurs in older adults, with variable durations of ACE inhibitor exposure, and typically resolves after cessation of the offending agent with supportive management [2,6,7,8]. In our case, a 77-year-old man, who had been taking an ACE inhibitor for more than 20 years, presented with unilateral tongue and lip edema without airway compromise. The patient’s rapid recovery following intravenous fluids, corticosteroids, and antihistamines - despite no clinical evidence of allergic reaction or anaphylaxis - was consistent with previous reports [1,8]. While high-quality evidence supporting the efficacy of corticosteroids and antihistamines in bradykinin-mediated angioedema is lacking [9,10], they may still be used in the acute setting [1,9].

Its asymmetric nature often shifts clinical suspicion toward other causes, such as trauma, infection, or mass lesions. Maintaining a high index of suspicion - supported by a thorough medication history - is essential to ensure timely discontinuation of the ACE inhibitor and appropriate supportive care [2,6,7].

Although ACE inhibitor-induced angioedema most commonly involves the lips, face, or oropharynx, the tongue is a particularly significant site due to the risk of rapid airway compromise, with reports suggesting that between 13% and 22% of patients will require airway intervention [3,11].

The clinical context of our case was typical for ACE inhibitor-induced angioedema: the patient reported no other symptoms, and the absence of focal infection signs, structural abnormalities, or persistent swelling on follow-up after cessation of ACE inhibitor made alternative diagnoses such as tumors or localized infection unlikely. This observation underscores the importance of a thorough medication history and careful clinical evaluation in distinguishing atypical angioedema from other acute tongue pathologies, as well as emphasizes the necessity of discontinuing the offending drug and avoiding re-challenge. Our case aligns with others in the literature in both presentation and recovery, reinforcing that unilateral tongue involvement, although rare, is a recognized variant of ACE inhibitor-induced angioedema [1,2,6,7,8].

Overall, this case highlights several key learning points. A high index of clinical suspicion, particularly in patients on long-term ACE inhibitor therapy, is essential for timely recognition and discontinuation of the offending agent. Supportive management - including airway assessment and drug cessation - remains the cornerstone of treatment. Documentation of rare presentations such as unilateral tongue angioedema contributes to increased clinical awareness, allowing earlier diagnosis and reducing the risk of unnecessary interventions or delayed care [1-3,6,7].

## Conclusions

Heightened awareness of ACE inhibitor-induced angioedema and its atypical presentations is crucial - not only to prompt appropriate supportive treatment and identify early signs of airway compromise but also to avoid unnecessary interventions, thereby improving patient safety. By documenting an exceptionally rare unilateral presentation of an already uncommon adverse reaction, this case report adds valuable insight to the literature and reinforces the importance of considering ACE inhibitors as a possible cause of isolated tongue swelling.
